# Identification of a New Marine Bacterial Strain SD8 and Optimization of Its Culture Conditions for Producing Alkaline Protease

**DOI:** 10.1371/journal.pone.0146067

**Published:** 2015-12-30

**Authors:** Hongxia Cui, Muyang Yang, Liping Wang, Cory J. Xian

**Affiliations:** 1 College of Environmental and Chemical Engineering, Yanshan University, Qinhuangdao, 066004, China; 2 Hebei Province Key Laboratory of Applied Chemistry, Qinhuangdao, 066004, China; 3 Sansom Institute for Health Research, School of Pharmacy and Medical Sciences, University of South Australia, Adelaide, 5001, Australia; University of Gdansk, POLAND

## Abstract

While much attention has been given to marine microorganisms for production of enzymes, which in general are relatively more stable and active compared to those from plants and animals, studies on alkaline protease production from marine microorganisms have been very limited. In the present study, the alkaline protease producing marine bacterial strain SD8 isolated from sea muds in the Geziwo Qinhuangdao sea area of China was characterized and its optimal culture conditions were investigated. Strain SD8 was initially classified to belong to genus *Pseudomonas* by morphological, physiological and biochemical characterizations, and then through 16S rDNA sequence it was identified to be likely *Pseudomonas hibiscicola*. In addition, the culture mediums, carbon sources and culture conditions of strain SD8 were optimized for maximum production of alkaline protease. Optimum enzyme production (236U/mL when cultured bacteria being at 0.75 mg dry weight/mL fermentation broth) was obtained when the isolate at a 3% inoculum size was grown in LB medium at 20 mL medium/100mL Erlenmeyer flask for 48h culture at 30°C with an initial of pH 7.5. This was the first report of strain *Pseudomonas hibiscicola* secreting alkaline protease, and the data for its optimal cultural conditions for alkaline protease production has laid a foundation for future exploration for the potential use of SD8 strain for alkaline protease production.

## Introduction

Over the years, researchers around the world have been interested in producing biological products particularly enzymes owing to their wide ranges of physiological, analytical and industrial applications. Among all biological resources for enzyme production, microorganisms are especially important because of their extensive biochemical diversity, possibility of mass culture and ease of genetic operations. So far, microorganisms are now known to play a key role in the production of both extracellular and intracellular enzymes in the commercial scale, and more than 3000 different microbial extracellular enzymes have been reported [1]. Among all the enzymes, proteases have occupied an important place as they were the first to be produced in bulk, and now they constitute about two-thirds of total enzymes used today [2], and the proteases are the main enzyme produced by microbial sources. They are used in a wide range applications, including in food, meat and leather processing industries as well as pharmaceutical industries. In particular, microbial alkaline proteases have dominated the worldwide enzyme market, accounting for a 67% share of the detergent industry [3,4].

A wide range of microorganisms was found to produce alkaline protease, including bacteria, molds, yeasts and mammalian tissues [5,6]. However, bacteria were preferred as they grow rapidly, need less space, could be easily maintained and were accessible for genetic operations. Species of *Bacillus*, *Pseudomonas*, *Halominas*, *Arthrobacter* and *Serratia* were the important protease producing bacteria. Among all bacterial species, bacilli played an important role in production of alkaline protease owing to their chemoorganotrophic characteristics and their abilities to secrete a high level of alkaline protease. In particular, more and more attention had been given to marine microorganisms of a wide range of habitats as enzymes derived from them were found relatively more stable and active than those derived from plants or animals [7, 8] and would have more advantages than traditional enzymes [9]. While alkaline (serine) proteases were found active over broad ranges of temperatures (35–80°C) and pH (7–12) [10], alkaline protease produced by marine bacteria had significant activity and stability at high pH and temperatures [11, 12].

Production of extracellular proteases of microorganisms was known to be largely influenced by the presence of easily metabolizable sugars (such as glucose) and medium components [13]. In addition, several other factors such as aeration, inoculum density, pH, temperature and incubation time also could affect the amount of protease produced [14–16]. However, the studies on alkaline protease production from marine microorganisms have been very limited [17]. In our recent study, one type of microorganism producing alkaline protease was isolated from sea muds of the Qinhuangdao sea area in China [18]. In the current study, we carried out further morphological, physiological and biochemical characterization, as well as 16S rDNA sequence analysis of this isolate SD8. In addition, we optimized its culture parameters for enhanced production of stable alkaline protease which was previously found to be stable in organic solvent and sodium dodecyl sulfonate (SDS) [18].

## Materials and Methods

This in vitro study did not involve humans, human data or animals, and thus there were no ethics or consent requirements for this study. As small samples of sea muds did not damage marine environment and wildlife and did not involve endangered or protected species, specific permission was not required for this work.

### Reagents and fermentation media

Casein used for the protease assay was bought from Sigma (St. Louis, MO, USA). The other chemicals used in the study were of analytical grade commercially available in China. All the experiments were carried out independently in triplicates and repeated twice.

The inoculum was prepared by adding one loop full of pure culture into 15 ml of sterile LB medium and incubated at 37°C on a rotary shaker (150rpm) for 24h. A 5% inoculum size was added to various protease producing culture media: (1) LB medium containing (g/L): peptone, 10; yeast extract, 5; NaCl, 10; pH 8.5; (2) starch medium containing (g/L): soluble starch, 20; beef extract, 5; peptone, 10; NaCl, 5; pH 8.5; (3) beef extract-peptone medium containing (g/L): peptone, 10; beef extract, 3; NaCl, 5; pH 8.5; and (4) glucose medium containing (g/L): peptone, 5; glucose, 5; K_2_HPO_4_, 2, pH 8.5. After incubation for 48 h at 37°Cwith shaking (150rpm), the cultures were harvested by centrifugation at 10000 rpm for 10 min at 4°C. The liquid supernatants were used as crude enzyme samples for measuring protease activity.

### Morphological, physiological, and biochemical characterization of the SD8 isolate

The bacterial strain SD8 known to produce an alkaline protease that was stable in SDS and organic stable used in the present study, which was isolated from sea muds of the sea area of Qinhuangdao, China [18]. This had been compared and identified tentatively as *Pseudomonas hibiscicola* [19] according to Bergey’s Manual of Determinative Bacteriology [20]. Morphological examination was carried out either on nutrient agar or in nutrient broth plus aged sea water, followed by Gram staining. Physiological and biochemical tests were carried out as described previously [21].

Further characterization was done on the basis of 16S rDNA sequencing as follows. The total genomic DNA of the strain SD8 was separated and purified by using the method described by Redburn and Pate [22]. The 16S rDNA of the isolate was amplified using the universal primers P1 (5′-AGAGTTTGATCATCCTGGCTCAG-3′) and P2 (5'-ACGGCTACCTTGTTACGACTT3′) [23]. The amplification was done by initial denaturation at 94°C for 3 min followed by 35 cycles of 94°C for 30s, 51°C for 30s, 72°C for 3 min and final extension at 72°C for 10 min. The PCR products were sequenced by Beijing Sun Biotech Co. Ltd (Beijing, China).

Sequence alignments of the strain SD8 were achieved with the NCBI’s BLAST program. All the sequences of 16S rDNA were aligned using the multiple sequence alignment program CLUSTAL-W (Dublin, Ireland). Phylogenetic and molecular evolutionary analyses were processed through the molecular evolutionary genetics analysis software MEGA 5.05 (Tempe, Arizona, USA).

### Experimental culture conditions on protease production

The current study has investigated optimal culture conditions for alkaline protease production from the SD8 isolate. To measure the effect of carbon sources on enzyme production, different carbon sources (sucrose, soluble starch, maltose, lactose, glycerin and glucose) were examined in the enzyme production media [24]. To examine the time kinetics of enzyme secretion, after the strain SD8 was inoculated in protease-producing LB medium and incubated at 37°C under shaking conditions (150 rpm), culture samples were withdrawn aseptically every 6 h and enzyme activity was monitored as described below. The growth curve of the strain SD8 was also investigated in LB medium at 37°C under shaking conditions (150 rpm). Optical density (OD600) was determined every 6 h. In order to investigate the influence of pH on protease production, the isolate was cultivated in LB medium at varying pH values (5.5–10.5, with the interval of increase of 1.0), and protease activity was quantified after incubation of 48 h at 37°C under shaking conditions at 150 rpm. To observe the effect of temperature on the protease production of the bacterial strain, 30°C and 37°C were selected for the culture. In addition, to investigate the effect of dissolved oxygen levels on the alkaline protease production, 10, 15, 20, 25, and 30 mL of culture liquid were enclosed to 100mL Erlenmeyer flasks, respectively, and alkaline protease activities were quantified after incubation with the optimal conditions revealed. Finally, in order to investigate the effect of inoculum size on alkaline protease secretion, 1%, 3%, 5% and 7% inoculum sizes were transferred to the culture media, respectively, and alkaline protease activities were detected after incubation with the other optimal conditions revealed above. The amount of bacteria in the optimum fermentation condition was weighed by an electronic balance after being dried for 5h at 80°C.

### Protease activity assay

A modification of the method of Kunitz [25] was used to assay protease activity using casein as a substrate and L-tyrosine as a standard. The 0.6ml reaction mixture consisted of 150μl of 1% casein in 200mM glycine-NaOH buffer (pH 10.0) and 150μl of culture supernatant. The reaction was started by adding culture supernatant at 40°C. After incubation for 15min, the reaction was stopped by adding 300μl of 0.4M trichloroacetic acid. The reaction liquid was kept on ice for another 10 min, then centrifuged at 10000 rpm for 10 min at 4°C. The 0.3ml supernatant was mixed with 1.5ml of a 0.4M Na_2_CO_3_ solution and 0.3ml of Folinphenol reagent and was incubated at 40°C for 20min. The concentration of L-tyrosine of digested casein was determined by monitoring an increase in absorbance at 680nm. The culture medium without SD8 bacteria added was used as the blank control in the absorbance reading. The calibration curve was constructed using L-tyrosine as a standard. One unit of protease activity was defined as the amount of enzyme that releases 1μg/ml of L-tyrosine equivalent per min [18].

### Statistics

All data were expressed as the means ± SEM. A one-way ANOVA using SPSS 13.0 software was used to conduct a statistical comparison of differences among the groups and a value of P<0.05 was considered as being statistically significant.

## Results

### Identification of strain SD8 that produces alkaline protease

**Fig 1:**
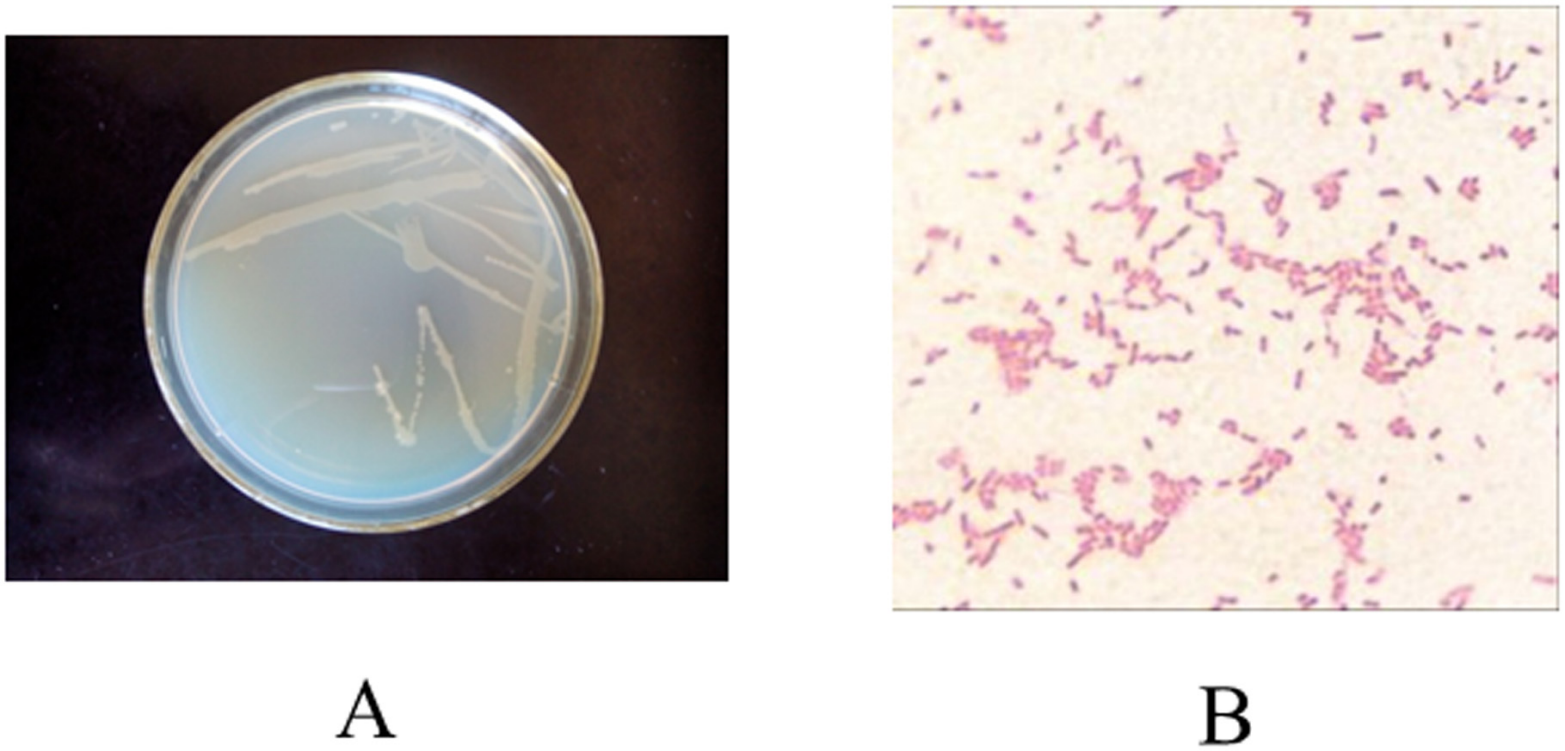
The colony morphology (A) and gram stain (B) of murine bacteria strain SD8.

The strain SD8 was characterized as a Gram negative, motile, rod-shaped bacterial strain. Its morphological and biochemical characteristics were listed in Table 1. The colony morphology and gram stain of strain SD8 were showed in Fig 1. Sugar using was positive except for glycerin. Voges Proskauer and Citrate were positive and other biochemical test results were negative. Based on these characteristics, the strain SD8 was identified probably as genus *Pseudomonas*.

**Table 1:** Morphological and biochemical characteristics of strain SD8.

	Results
**Colony morphology**	Round, yellow, apophysis, marginal tidy, smooth surface, mucoid
**Gram stain**	-
**Motility**	+
**Shape**	Rod
**Glucose**	+
**Manntitol**	+
**Sucrose**	+
**Fructose**	+
**Glycerin**	-
**Starch**	+
**Maltose**	+
**Lactose**	+
**Indole**	-
**Methyl red**	-
**VogesProskauer**	+
**Citrate**	+
**Urease**	-
**H**_**2**_ **S production**	-
**Amylolysis**	-
**Gelatin hydrolysate**	-

“+”: positive; “-”: negative.

**Fig 2:**
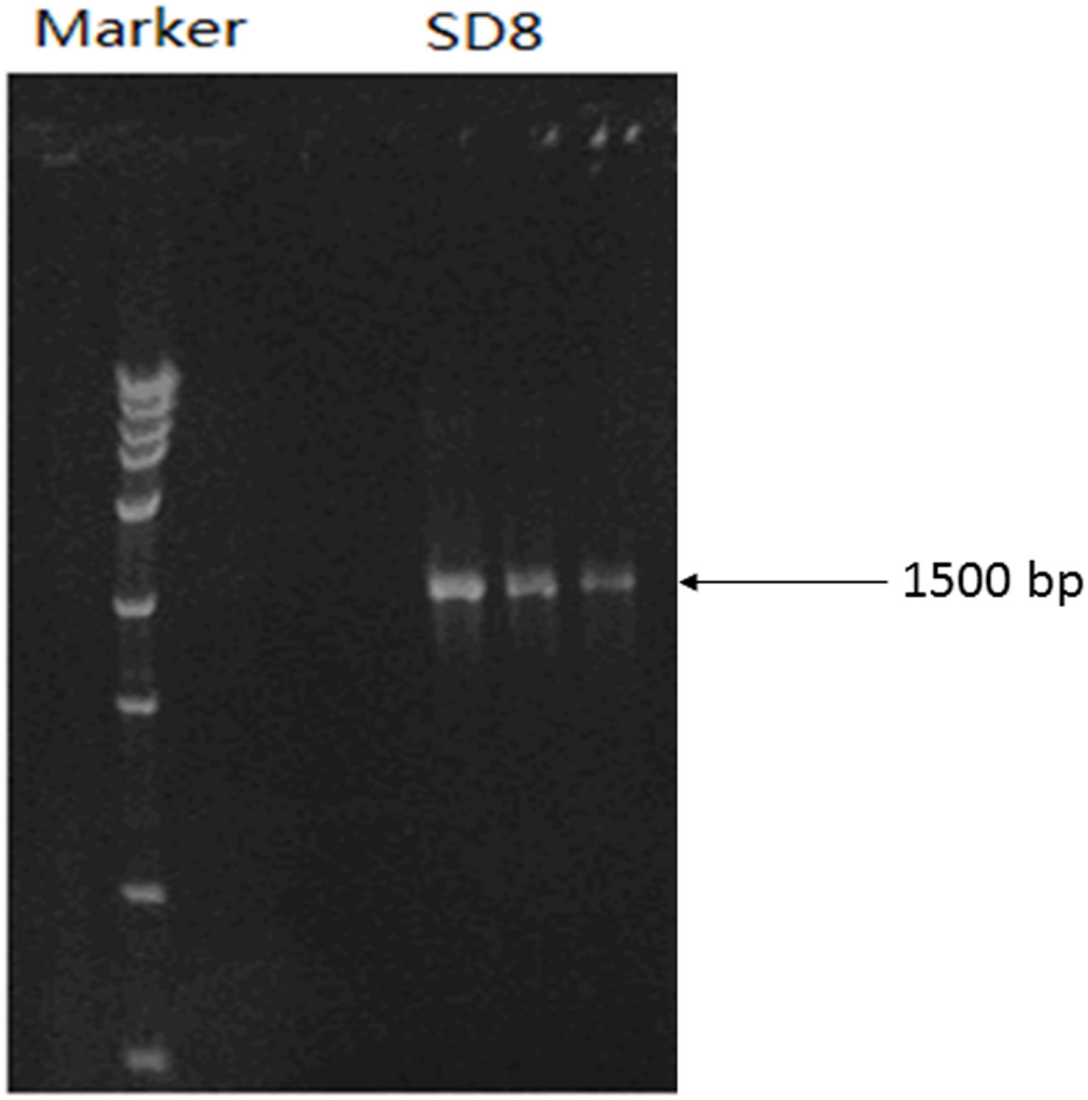
Electrophoregram of PCR products of strain SD8.

To carry out 16S rDNA sequence analysis, the genome of strain SD8 was used for PCR amplification of 16S rDNA. Agarose gel electrophoresis of PCR product was shown in Fig 2. It can be seen that the molecular weight of PCR product of strain SD8 was corresponding to 1500bp of the DNA marker and the amplification was successful. The DNA sequence of 1440 bp product was obtained by 16S rDNA sequencing (Beijing Sanpo Polygala Biological Technology LTD, Beijing, China). The gene sequence was uploaded to GenBank database (accession number KM668099).

**Fig 3:**
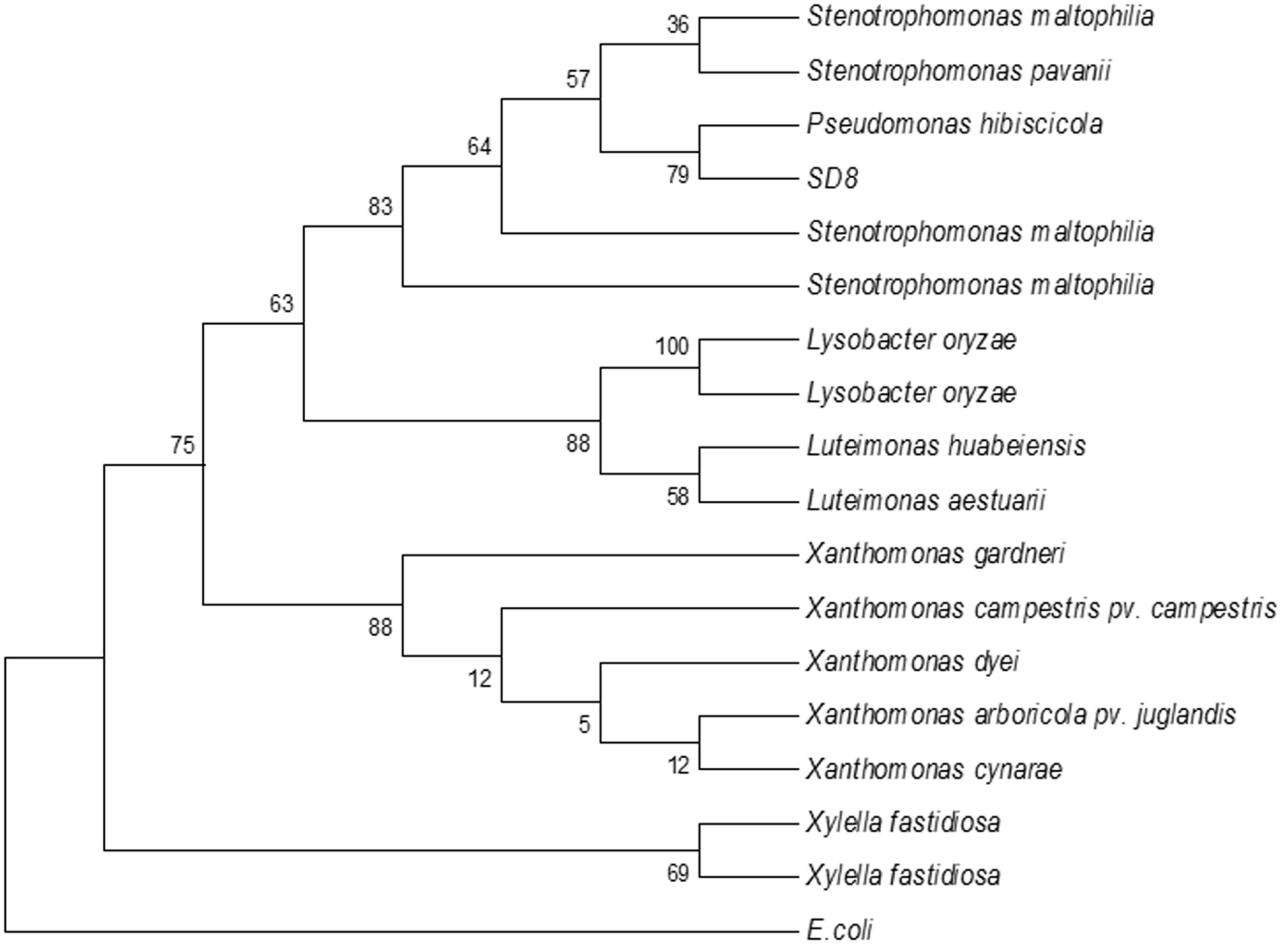
Phylogenetic tree analysis of strain SD8 based on comparison of its 16S rDNA sequence with those of closely related strains. The sequence of the 16S rDNA of *E*. *coli* was used as an outgroup. The tree was generated using the neighbor-joining v method.

Following the sequence determination of the PCR product and 16S rDNA analysis, the strain SD8 was phylogenetically characterized and identified/compared with the closest relatives using BLAST (NCBI) search. Sequence analysis of 16S rDNA sequence for the isolate SD8 based on in silico analysis showed a 99% homology with *Pseudomonas hibiscicola* (Fig 3). The strain SD8 falls in the cluster comprising members of the *Pseudomonas hibiscicola* with the reliability of 79%. Thus with this evidence of identification, the isolate may belong to *Pseudomonas hibiscicola*.

### Effect of fermentation medium on alkaline protease production

**Fig 4:**
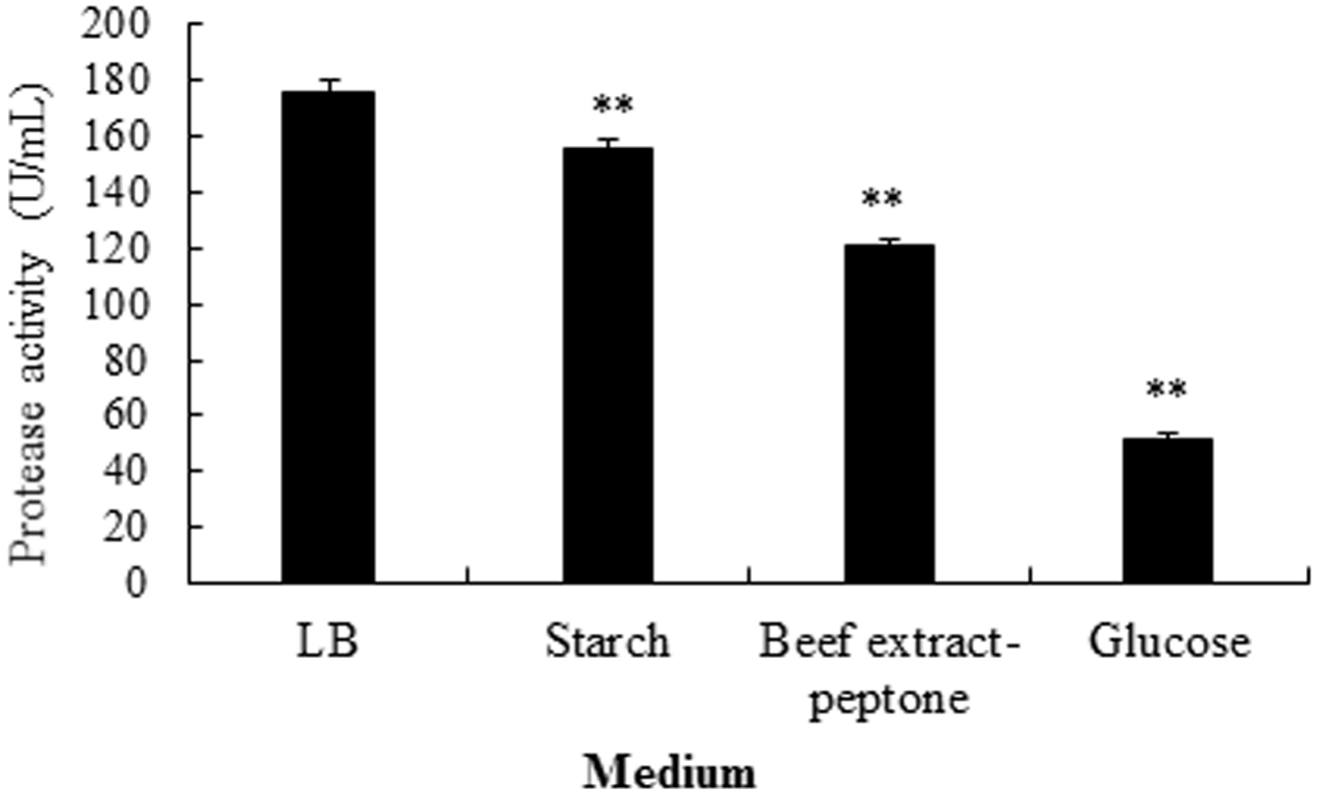
Effects of medium types in influencing the production of alkaline protease from strain SD8. The strain was cultured 48 h at 37°C. *P<0.05 and **P<0.01 compared to LB medium. All the data were given as means ± SEM (n = 3).

Four mediums were selected to observe the medium effect on the protease production of strain SD8. Among the four media, the LB medium was found to produce the alkaline protease with the highest activity (up to 176 U/mL) (Fig 4). The enzyme activities yielded from the starch (P<0.05), beef extract-peptone (P<0.01) and glucose medium (P<0.01) were significantly lower than that from the LB medium. Hence, the LB medium was selected to carry out the subsequent experiments.

**Fig 5:**
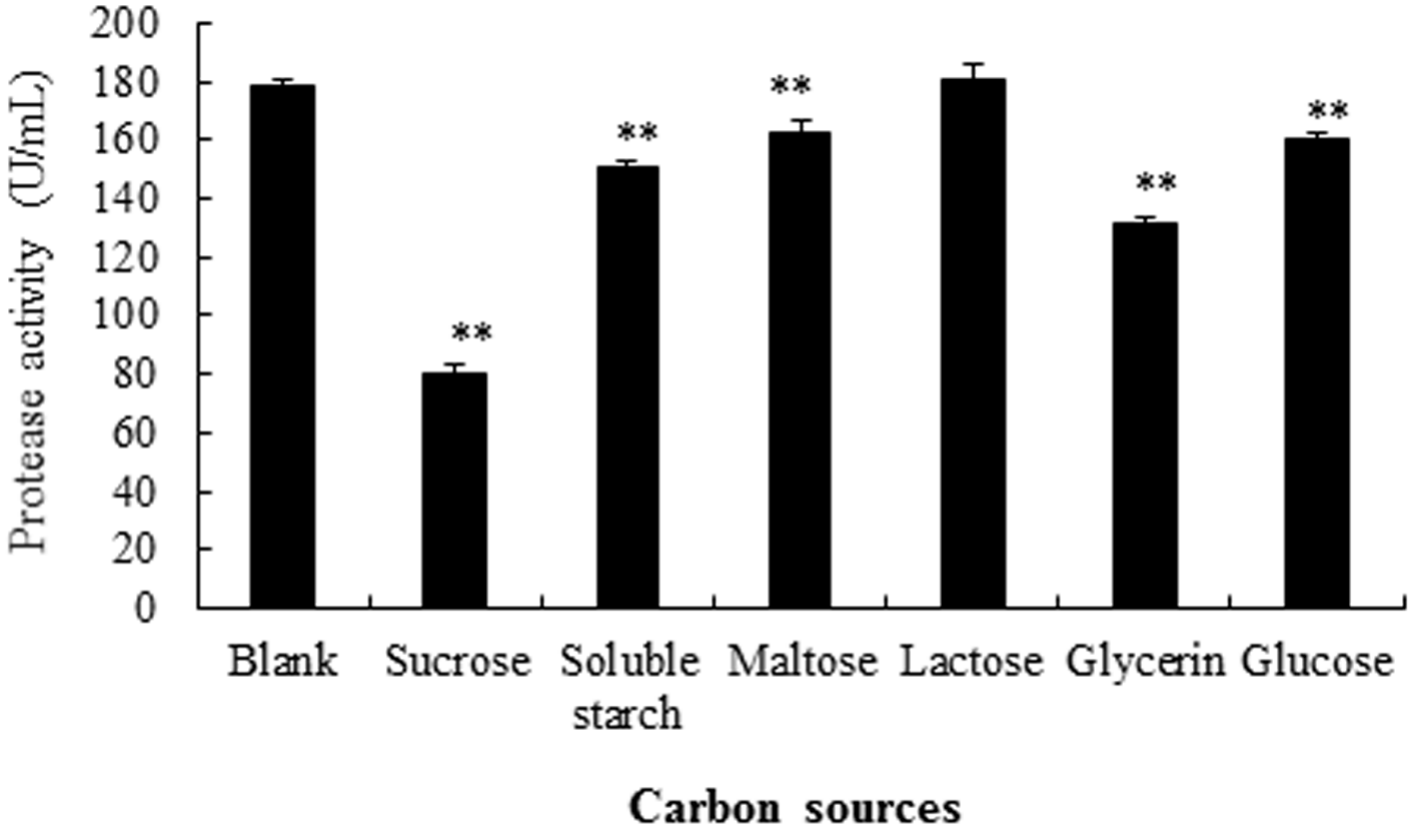
Effects of carbon sources in influencing the production of alkaline protease from strain SD8. LB medium without carbon added was selected as blank control. *P<0.05 and **P<0.01 compared to blank control. All the data were given as means ± SEM (n = 3).

Previously, different carbon sources were found to have different influences on extracellular enzyme production [26]. In the current study, for investigating the influence of carbon sources on production of alkaline protease, effects of adding 5% carbon from different sources to the LB medium were examined (Fig 5). When compared to LB medium alone (blank control, without additional carbon added), while addition of lactose did not affect the alkaline protease activity, addition of maltose or glucose (P<0.05), or either of the three other carbon sources (sucrose, soluble starch, and glycerin) (P<0.01) significantly decreased the enzyme activity when compared to the blank control. The alkaline protease activity was found the lowest when sucrose was added (reduced about 50%). Therefore, the LB medium without carbon source was selected to perform the subsequent experiments.

### Effect of culture conditions on alkaline protease production

**Fig 6:**
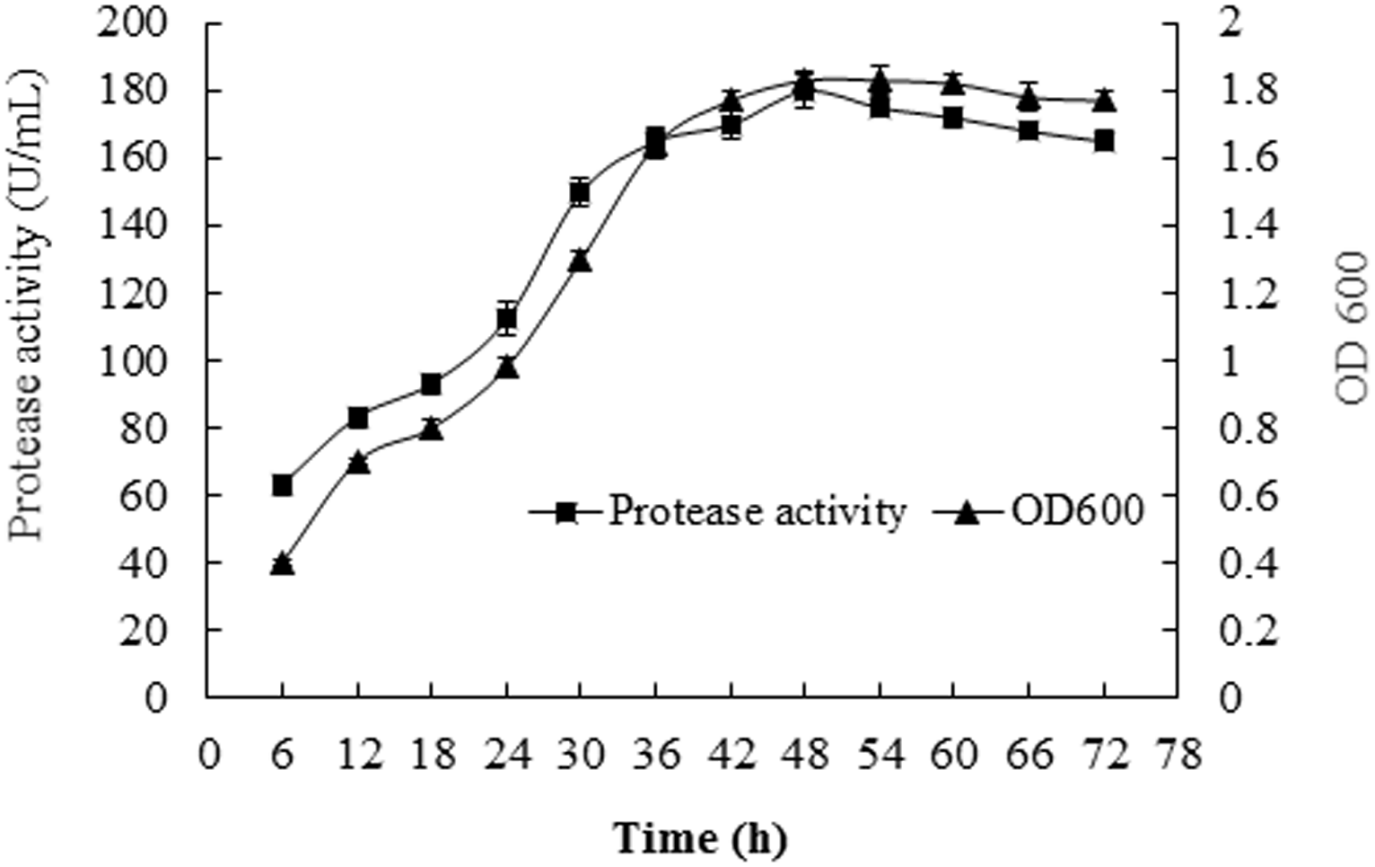
Effects of culture time in influencing the production of alkaline protease and growth from strain SD8. The optimum medium was used. All the data were given as means ± SEM (n = 3).

The current study has investigated the effect of incubation time on alkaline protease production of strain SD8. Protease activity was found to increase rapidly after incubation for 24 h (Fig 6), and it was highest (180 U/mL) when the incubation time was 48 h, after which time it declined slowly. Compared to 48 h, all other incubation time periods produced significantly lower yields of alkaline protease. Therefore, the optimal fermentation time for alkaline protease production was 48 h for strain SD8. The growth curve of strain SD8 was also showed in Fig 6. The amount (as assessed by OD600 measurement) of strain SD8 increased quickly after 18 h, and it reached up to 1.77 at 42 h, which was almost to the same as the maximum (1.83) at 48 h. After that, the amount of strain SD8 declined slowly.

**Fig 7:**
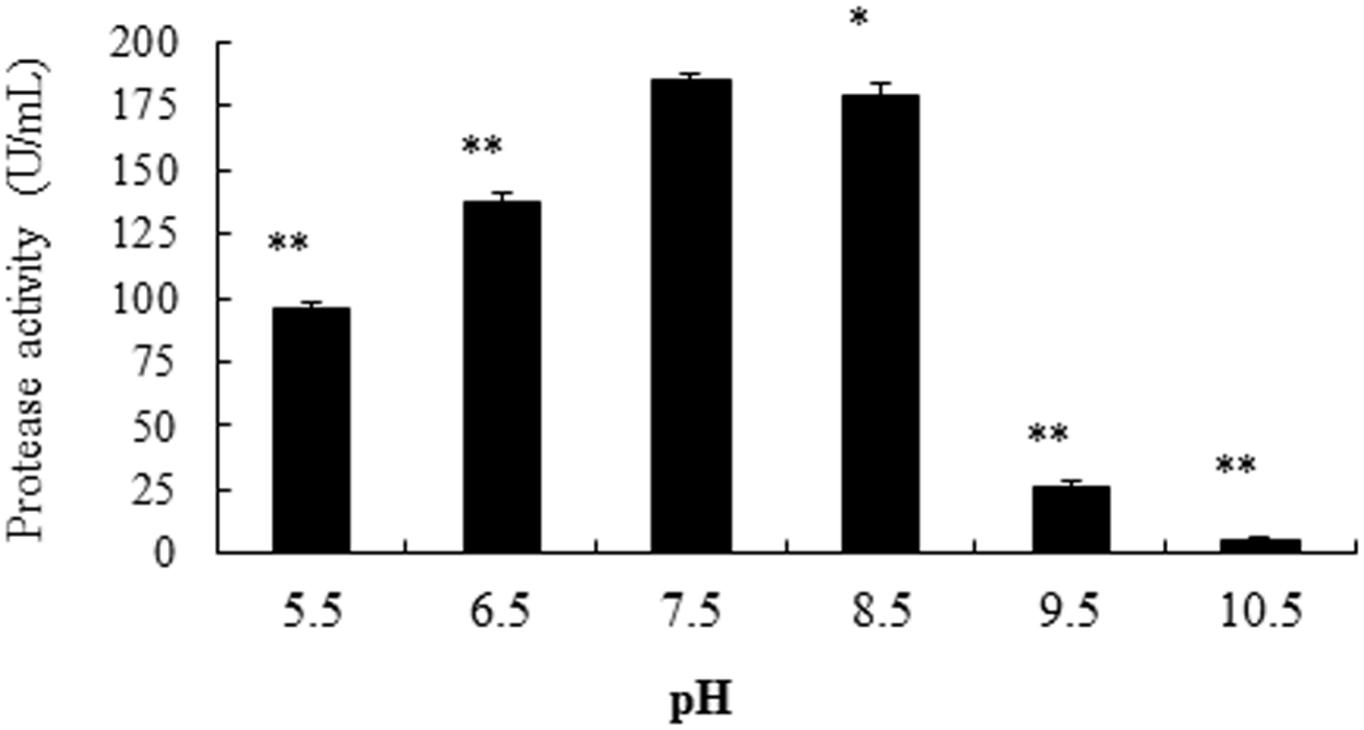
Effects of initial pH in influencing the production of alkaline protease from strain SD8. The single factor investigated was selected at optimal conditions. *P<0.05 and **P<0.01 compared to pH 7.5. All the data were given as means ± SEM (n = 3).

The current study also examined influences of culture medium pH and temperatures and found a gradual increase in the protease production in strain SD8 with increasing pH, with the optimum being at pH 7.5 (185U/mL) (Fig 7). At pH 8.5, the protease activity was 179 U/mL, which was lower than that of pH 7.5 (P<0.05). In either lower pH (5.5 or 6.5) or higher pH (9.5 or 10.5) cultures, the protease activity was significantly lower than that of pH 7.5 (P<0.01). When the incubation temperatures were 37 and 30°C, the alkaline protease activities of the cultures were 185 and 216 U/mL, respectively. Thus, 30°C was chosen for carrying out the subsequent work.

**Fig 8:**
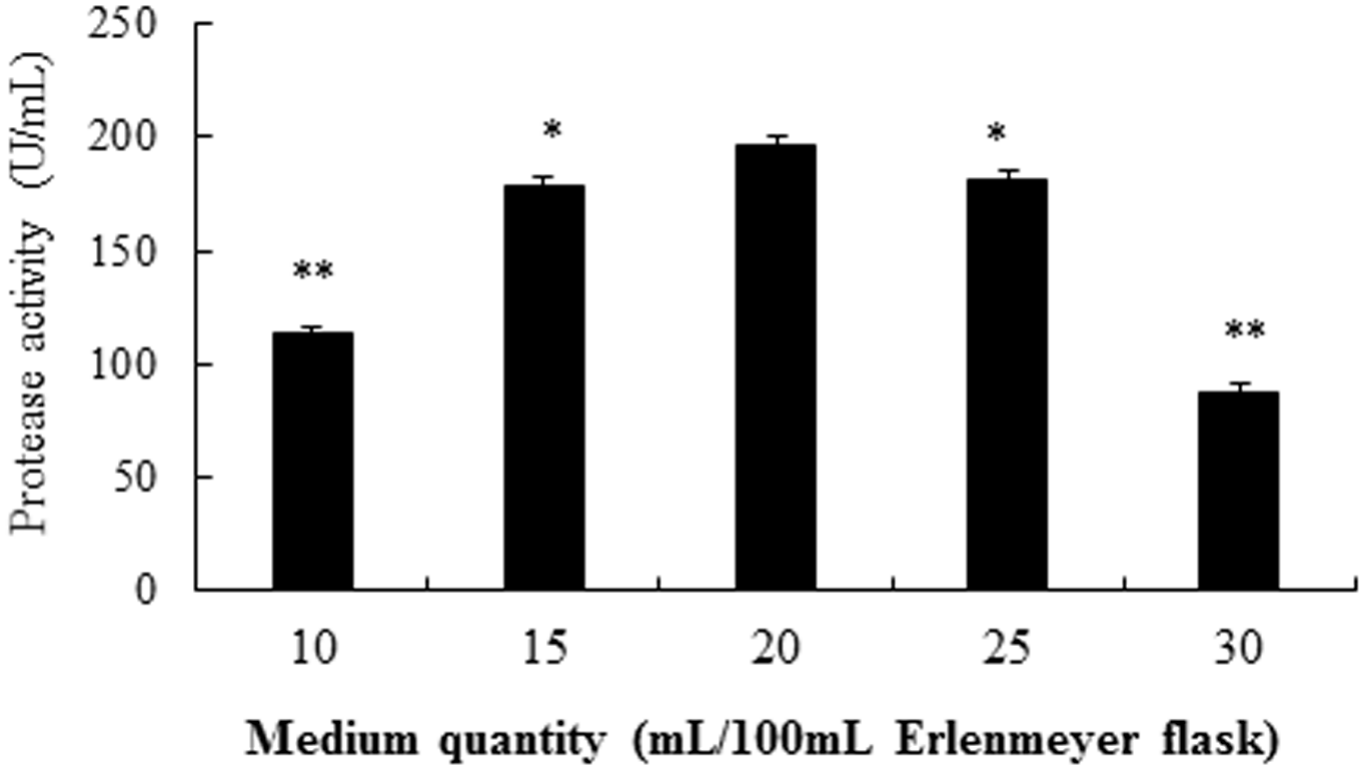
Effects of medium quantity in influencing the production of alkaline protease from strain SD8. The single factor investigated was selected at optimal conditions. *P<0.05 and **P<0.01 compared to 20 mL/100 mL Erlenmeyer flask. All the data were given as means ± SEM (n = 3).

Different dissolved oxygen levels in the incubation liquid of the bioreactor could be obtained by varying medium quantity in the Erlenmeyer flask, which could influence the alkaline protease production. As shown in Fig 8, the alkaline protease activity was highest (196 U/mL) with the 20 mL culture liquid when under the optimal conditions established above. When the culture medium was 15 or 25 mL, the respective alkaline protease activity was significant lower than that of 20mL (P<0.05). When the medium was even lower or higher in volume (10 or 30 mL), the respective alkaline protease activity was significantly and substantially lower than that of 20 mL (P<0.01).

**Fig 9:**
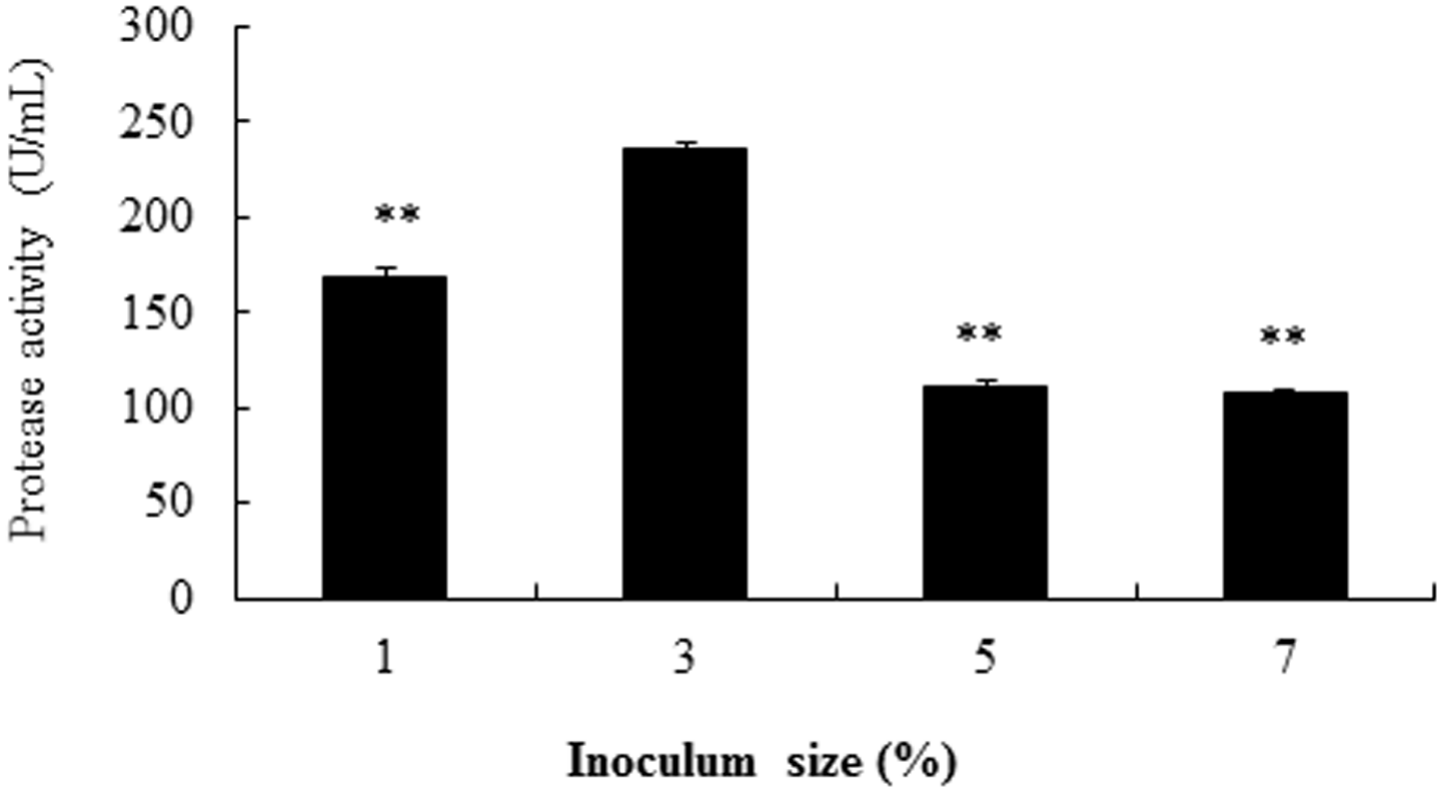
Effects of inoculum size in influencing the production of alkaline protease from strain SD8. The single factor investigated was selected at optimal conditions. **P<0.01 compared to 3% inoculum size. All the data were given as means ± SEM (n = 3).

The organism density (as affected by the inoculum size) could also affect the alkaline protease production in strain SD8. The enzyme activity was highest (236 U/mL) with the 3% inoculum size. When the inoculum size was lower (1%) or higher (5 or 7%), the alkaline protease activity was significantly lower when compared to the 3% inoculum size (P<0.01, Fig 9). So 3% inoculum size was chosen as the optimal condition.

The bacterial mass was also determined in optimum fermentation conditions. The bacterial mass was found to be about 0.75 mg dry weight/mL fermentation broth when producing the optimum protease activity.

## Discussion

More attention has been given to marine microorganisms for enzyme production as enzymes derived from them have been found in general relatively more stable and active compared to those from plants and animals. While alkaline protease produced by marine bacteria is known to have significant activity and stability at high pH and temperatures, studies on alkaline protease production from marine microorganisms have been very limited. In this study, one marine bacterial strain SD8 isolated from sea muds of the Qinhuangdao sea area in China producing alkaline protease relatively at a low yield was identified to be likely *Pseudomonas hibiscicola* following morphological, physiological and biochemical characterization as well as 16S rDNA sequence analysis. In addition, the current study has identified the LB medium (among 4 different media) as the optimum culture medium producing alkaline protease by the strain SD8. Furthermore, the current study has determined the optimal culture conditions for talkaline protease production (no additional carbon source, 48h fermentation time, initial pH7.5, 30°C temperature, 20 mL culture liquid/100 mL Erlenmeyer flask and 3% inoculum size). When cultured at the optimized parameters, alkaline protease production was enhanced to 236 U/mL.

The strain SD8 was identified as *Pseudomonas hibiscicola* based on data of its morphology, physiology and biochemistry assays as well as 16S rDNA sequence analyses. While there were some reports that genus *Pseudomonas* could produce alkaline protease including *Pseudomonas aeruginosa* [27], *Pseudomonas fluorescens* [28] and *Pseudomonas putida* [29], *Pseudomonas hibiscicola* was not found to secrete alkaline protease up to now. The finding of the strain SD8 being able to produce the alkaline protease and belonging to *Pseudomonas hibiscicola* has thus enriched our understanding of the characteristics of the *Pseudomonas hibiscicola*.

Since the types of medium and carbon sources could influence the production of alkaline protease [24], the current study has carried out the optimization of medium and carbon sources. It was found that the production of alkaline protease of strain SD8 was substantially different with different types of medium and carbon sources, with SB medium without additional carbon added being found to be optimal. Kumar et al [5] reported that lactose was the best carbon source for protease production by *Marinobacter* sp. GA CAS9, and Pant et al [6] reported that galactose was the best carbon source for protease production by *Bacillus subtilis*. These and our current studies have proved that the carbon sources affect the protease production and the best carbon source is different for different bacterial strains.

In addition, sine the culture conditions also could influence the production of alkaline protease of microorganisms [16], the current study has also investigated optimal culture conditions of SD8 strain for the production of alkaline protease. The results indicated that the culture time, initial pH, temperature, medium quantity, and inoculum size could also influence the production of alkaline protease. The phenomenon of the effect of initial pH on production of alkaline protease was similar as that reported previously [30]. Our data of the optimal cultural conditions for alkaline production from SD8 strain has laid a foundation for further exploring the potential use of SD8 strain for alkaline protease production.

The activity of alkaline protease by the strain SD8 was found lower than that by *Marinobacter* sp. GA CAS9 or by *Bacillus subtilis* [5, 6, 31]. While the *Marinobacter* sp average activity was somewhere in the range of 400–1000 U/ml [5] and that of *Bacillus subtilis* was between 576–842 U/ml [31] at 24–48 hours, that of SD8 strain (or *P*. *hibiscicola*) was 236 U/ml at 48 hours. However, the alkaline protease produced by SD8 has been recently found to be stable in alkaline and SDS solutions and organic solvents [18]. These characteristics of the alkaline protease produced by SD8 will be important for its potential applications in industries that require a stable protease, as these extreme conditions often exist in commercial processes.

## Conclusions

In this study, marine bacterial strain SD8 was initially classified to belong to genus *Pseudomonas* by morphological, physiological and biochemical characterizations, and then identified to be likely *Pseudomonas hibiscicola* through 16S rDNA sequence. In addition, in attempts to optimize its culture conditions, this study has investigated mediums, carbon sources and culture conditions for maximum production of alkaline protease. Optimum enzyme production (236U/mL with bacterial mass being at 0.75 mg dry weight/mL fermentation broth) was obtained when the isolate at a 3% inoculum size was grown in LB medium at 20 mL medium/100mL Erlenmeyer flask for 48h culture at 30°C with an pH 7.5. This was the first report of strain *Pseudomonas hibiscicola* secreting alkaline protease. The finding of the strain SD8 being able to produce the alkaline protease and belonging to *Pseudomonas hibiscicola* could enrich our understanding of the characteristics of the *Pseudomonas hibiscicola*, and our data for its optimal cultural conditions for alkaline protease production has laid a foundation for future exploration for the potential use of SD8 strain for alkaline protease production. Further studies will be required to investigate the production, activity and stability of the alkaline protease produced by SD8 strain and conduct comparative studies with proteases produced by other species or bacterial strains.
